# Obesity Pharmacotherapy: Current Perspectives and Future Directions

**DOI:** 10.2174/157340313805076322

**Published:** 2013-02

**Authors:** Monika Misra

**Affiliations:** Department of Pharmacology, Jawaharlal Nehru Medical College, Aligarh Muslim University, Aligarh, Uttar Pradesh: 202002, India

**Keywords:** Obesity, Pharmacotherapy, Drug targets.

## Abstract

The rising tide of obesity and its related disorders is one of the most pressing health concerns worldwide, yet existing medicines to combat the problem are disappointingly limited in number and effectiveness. Recent advances in mechanistic insights into the neuroendocrine regulation of body weight have revealed an expanding list of molecular targets for novel, rationally designed antiobesity pharmaceutical agents. Antiobesity drugs act via any of four mechanisms: 1) decreasing energy intake, 2) increasing energy expenditure or modulating lipid metabolism, 3) modulating fat stores or adipocyte differentiation, and 4) mimicking caloric restriction. Various novel drug candidates and targets directed against obesity are currently being explored. A few of them are also in the later phases of clinical trials. This review discusses the development of novel antiobesity drugs based on current understanding of energy homeostasis

## INTRODUCTION

Obesity has progressed to epidemic proportions globally, with more than 1.6 billion adults overweight and at least 400 million of them clinically obese [1]. It is a leading contributor to the global burden of chronic diseases like type 2 diabetes, cardiovascular disease, hypertension, stroke, and certain forms of cancer. The health outcomes range from increased risk of premature death to serious chronic conditions that reduce the overall quality of life [1]. Each year, an estimated 300,000 adults in the United States die of causes related to obesity [2]

According to the guidelines of the National Institute of Health (NIH) regarding the treatment of obesity, “weight loss drugs approved by the FDA [US Food and Drug Administration] may only be used as part of a comprehensive weight loss program, including dietary therapy and physical activity, for patients with a BMI [body mass index] of ≥30 with no concomitant obesity-related risk factors or diseases, and for patients with a BMI of ≥27 with concomitant obesity-related risk factors or diseases”[3].The risk factors and diseases considered important enough to warrant pharmacotherapy at a BMI of 27--29.9 are hypertension, dyslipidemia, coronary heart disease (CHD), type 2 diabetes, and sleep apnea [3]. In 2007--2008, the prevalence of American adults having a BMI greater than 30 was 32.2% among men and 35.5% among women [4]. In light of the NIH guidelines, this startling preponderance of obesity indicates pharmacotherapy is warranted for more than one-third of all American adults. Yet, existing medicines to combat the problem are disappointingly few in number and efficacy. In the past few years, understanding of the mechanisms involved in regulating feeding behavior and energy expenditure has evolved considerably, and targeting these mechanisms for drug development has garnered greater attention as well. This review discusses the neuroendocrine control of energy homeostasis, drug targets, and the possible future range of anti-obesity agents.

## PATHOPHYSIOLOGY OF OBESITY

**Fig. (1):**
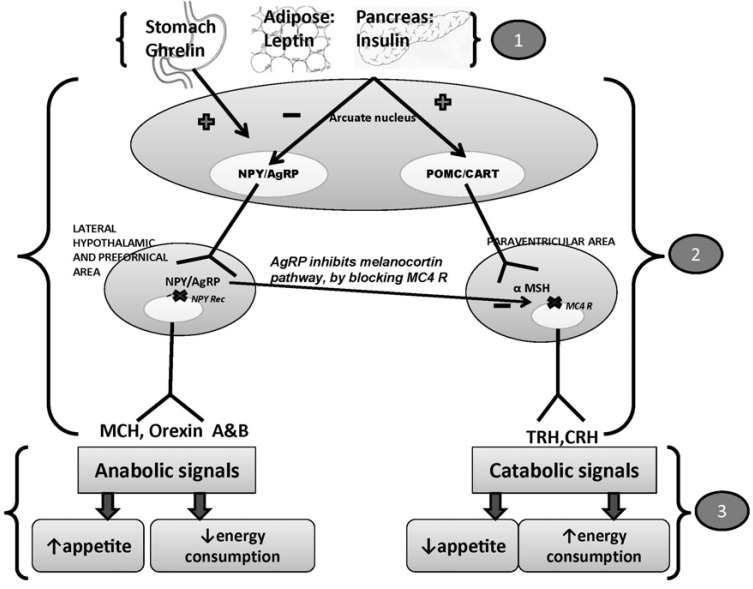
Neuroendocrine control of energy homeostasis (details in text). afferent system generates signals released from adipose tissue (leptin), pancreas (insulin) and stomach(ghrelin)central processing unit present in hypothalamus having two set of neurons: orexigenic and anorexigenic neuronsefferent system carries out anabolic and catabolic signals by modulating feeding behavior or energy expenditure NPY/AgRP= Neuropeptide Y/ Agouti related peptide; POMC/CART= Pro-opiomelanocortin/ Cocaine and Amphetamine Related Transcript; NPY Rec= Neuropeptide receptor; MCH= Melanin Concentrating Hormone; α MSH= α Melanocyte Stimulating Hormone; Mc4R= Melanocortin receptor; TRH= Thyrotrophin Releasing Hormone; CRH= Corticotrophic Releasing Hormone

The etiology of obesity is complex. Genetic, environmental, and psychological factors are implicated in its causation. Obesity is a disorder of energy balance. When food-derived energy chronically exceeds energy expenditure, the excess calories are stored as triglycerides in adipose tissue. Notwithstanding the marked fluctuations in daily food intake, body weight remains remarkably stable in most humans. In response to alterations in body adiposity, the brain triggers compensatory physiological adaptations that resist weight change. The two sides of the energy equation, i.e., consumption and expenditure, are finely regulated by neural and humoral mechanisms [5,6]. These neural and humoral mechanisms work in concert as the neurohumoral system to maintain body energy status. The neurohumoral system is comprised of the afferent pathway, the central processing unit, and the efferent pathway [5] (Fig. **1**).

**Fig. (2).**
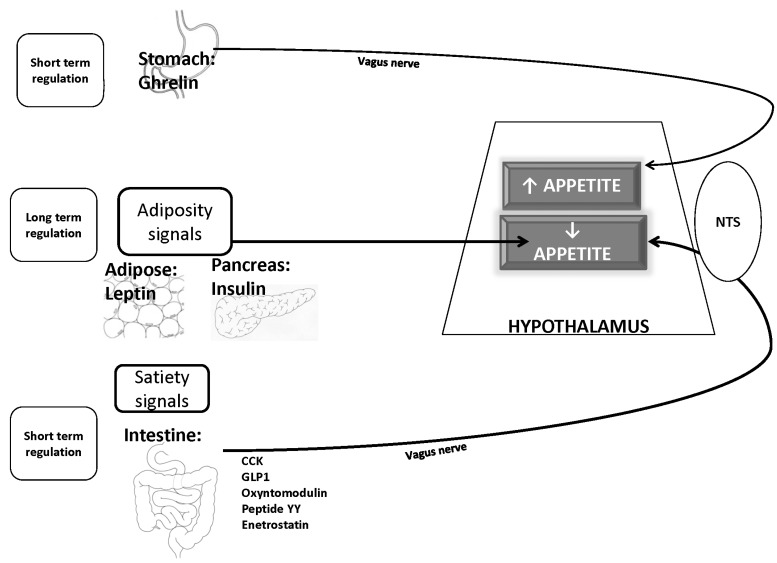
Short and long term regulation of energy intake (details in text) NTS= Nucleus tractus solitarius ; CCK= cholecystokinin; GLP1= Glucagon like peptide1

The afferent system generates humoral signals from adipose tissue (leptin), pancreas (insulin), and stomach (ghrelin). Acting in the brain, leptin and, to a lesser extent, insulin decrease food intake and increase energy expenditure, promoting weight loss. They are consequently termed catabolic adiposity signals. Impinging on the same neuronal targets, ghrelin exerts opposite effects and is thus an anabolic hormone. The amount of leptin is in direct proportion to adipose stores. Weight gain evokes proportionate increases in catabolic hormones and decreases ghrelin, whereas weight loss causes the opposite [5]. Long-term regulation of energy stores depends on adiposity signals like leptin and insulin, while ghrelin communicates short-term changes in nutritional status. Apart from ghrelin, many other gastrointestinal signals communicate short-term alterations in nutritional status to the brain via humoral and neural stimuli. A number of satiety signals that are released from the intestine include cholecystokinin (CCK), pancreatic polypeptide (PP), peptide-YY (PYY), oxyntomodulin, enterostatin, and glucagon -like peptide-1 (GLP-1) [5-10]. Another satiety signal, amylin, is cosecreted with insulin from the pancreas. These short-acting satiety and hunger signals are transmitted via afferent fibers of the vagus nerve to the caudal brainstem in nucleus tractus solitarius, from where they also influence the hypothalamus. The sensitivity of brainstem responses to afferent gastrointestinal signals is modulated by long-acting catabolic adiposity signals [5] (Fig. **2**).

The central processing unit is located primarily in the hypothalamus, especially in the arcuate nucleus, which integrates the afferent signals from leptin, insulin, and ghrelin. There are two major types of neurons in this locale that bear leptin receptors; these are anorexigenic neurons and orexigenic neurons. Pro-opiomelanocortin (POMC) and cocaine- and amphetamine-related transcript (CART) neurons are first-order anorexigenic neurons. These are activated by leptin and secrete α-melanocyte stimulating hormone (α-MSH), which acts on second-order neurons present in the paraventricular area that express melanocortin 4 receptor(MC4R). The signaling through these pathways induces expression of corticotrophic-releasing hormone (CRH) and thyrotrophin-releasing hormone (TRH) [5]. Neuropeptide Y (NPY)/ Agouti-related peptide (AgRP) neurons are the first-order orexigenic neurons. They produce NPY and AgRP, which are the initial targets of leptin action. NPY acts on second-order neurons in the lateral hypothalamic area and the perifornical area. The second-order neurons express orexigenic peptides that are melanin-concentrating hormone (MCH) and orexins A and B. The signaling through these neurons is increased by ghrelin and attenuated by leptin. AgRP exerts its anabolic effect by inhibiting the anorexigenic or melanocortin arm [5].

The efferent system carries out orders from the hypothalamic nuclei in the form of feeding behavior and energy expenditure [5]. Anorexigenic signaling through TRH and CRH induces anorexia and thermogenesis. On the other hand, the orexigenic signaling via orexigenic peptides (MCH and orexin A and B neuropeptides) results in increased food intake and decreased energy expenditure.

Several neurotransmitters (serotonin, noradrenaline, dopamine, and histamine), peptides (neuromedin U, urocortin, bombesin, amylin, galanin), hormones (thyroid hormone, growth hormone) and cytokines (ciliary neurotrophic factor) also play a role in modulating feeding behavior and energy expenditure [5-10].

## LEPTIN AND LEPTIN RESISTANCE

No discovery has had a greater impact on obesity research than that of leptin in 1994 [11]. Leptin is the product of OB gene, a 146 amino acid protein, secreted from white adipose tissue [12]. The gene encoding for leptin is mutated in genetically obese (ob/ob) mice. The gene encoding for the leptin receptor, a class I cytokine receptor, is mutated in fatty (fa/fa) rats and in diabetic (db/db) mice [12]. Leptin binds to its receptor and activates Janus kinase and STAT (signal transducers and activators of transcription) signal transduction pathway. STAT3 appears to be an important transcription factor recruited during signaling. It translocates to the nucleus and induces the production of anorectic neuropeptide POMC, while repressing the production of orexigenic neuropeptide AgRP. It also induces the expression of suppressor of cytokine signaling-3, which is a negative regulator of leptin signaling [13]. 

Leptin is an important hormone that maintains body weight by acting through a negative feedback mechanism of energy consumption and utilization. The finding that leptin deficiency in animals results in morbid obesity generated hope that exogenous administration of leptin would ameliorate obesity [11]. However, as common obesity is a state of leptin resistance, the exogenous administration of even exceedingly high doses of leptin has proven relatively ineffective at reducing body weight [14-16].

**Fig. (3).**
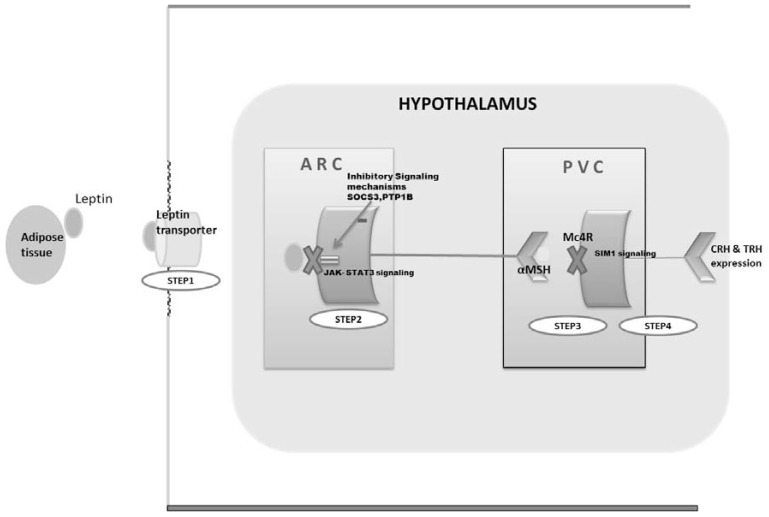
Strategies to overcome leptin resistance (Details in text) Step 1: Modified leptin bypassing normal brain transport or use of intranasal leptin Step 2: Stimulating leptin signaling: 
Activating STAT3, an important transcription factor recruited during leptin signaling  SOCS3 & PTP1B antagonists (inhibition of autoinhibitory factors involved in leptin signaling) Step 3: Melanocortin Mc4 receptor agonist (downstream pathway of leptin signaling exerting anorectic and thermogenic effect ) Step 4: Stimulating melanocortin signaling: SIM1 stimulation (a transcription factor that acts as a mediator for the anorectic, but not thermogenic effects of melanocortins) ARC= Arcuate nucleus; PVC= paraventricular nucleus; JAK= Janus kinase; STAT3=Signal transducers and activators of transcription; SOCS3= Suppressor of cytokine signaling-3; PTP1B= Protein tyrosine Phosphatase1 B; α MSH= α Melanocyte stimulating hormone; Mc4R= Melanocortin receptor; TRH= Thyrotrophin Releasing Hormone ;CRH= Corticotrophic Releasing Hormone; SIM1= Single- minded homologue 1.

In the past few years, insight into the mechanisms of leptin resistance has been elucidated. Leptin resistance results from impairments in leptin action at multiple levels. Leptin is usually transported across the blood-brain barrier by a specialized leptin-transporter that is impaired in obesity [17]. In mice, a high-fat diet has also been shown to impair leptin signaling by attenuating STAT3 activation, thus contributing to leptin resistance [18].Leptin receptor activation engages intracellular proteins, such as protein tyrosine phosphatase1 B (PTP1B) and suppressor of cytokine signaling-3, that terminate receptor signaling. Increased activity of these autoinhibitory factors attenuates leptin signaling and is also implicated in leptin resistance [17]. All of these factors are targeted in drug development in order to increase leptin sensitivity in obese individuals (Fig. **3**).

## PHARMACOTHERAPY OF OBESITY

**Fig. (4).**
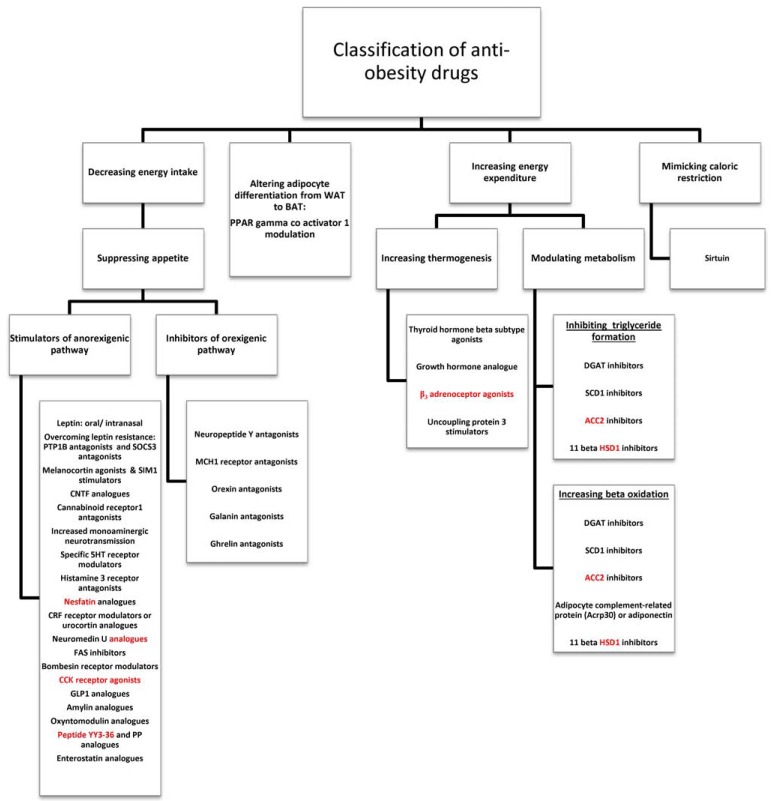
Classification of antiobesity drugs according to mechanism of action (details in text) WAT= White adipose tissue; BAT= Brown adipose tissue; PPAR= Peroxisome proliferator-activated receptors;SOCS3= Suppressor of Cytokine Signaling-3; PTP1B= Protein Tyrosine Phosphatase1B; SIM1= Single- minded homologue 1; CNTF= Ciliary neurotrophic factor; 5HT= serotonin; MCH= Melanin concentrating hormone; CCK= cholecystokinin; GLP1= Glucagon Like peptide1; PP= Pancreatic polypeptide; FAS=Fatty acid synthase; CRF= Corticotrophin releasing factor; DGAT= Diacylglycerol acyltransferase; SCD= Stearoyl CoA desaturase; ACC= Acetyl coA carboxylase; HSD= Hydroxysteroid dehydrogenase.

Obesity is a state of sustained disequilibrium between energy intake and energy expenditure. The pharmacotherapies for obesity are directed at the following: 1) decreasing energy intake (e.g. appetite suppressants and lipase inhibitors), 2) increasing energy expenditure, 3) modulating fat storage or adipocyte differentiation, or 4) mimicking caloric restriction [8,19] (Fig. **4**).

The history of the pharmacotherapy of obesity dates back to the 1930s. Many drugs used to treat obesity in the past have been discontinued because of their potential to be abused and their toxicities [20-27] (Table **1**). Rimonabant is a cannabinoid receptor 1 antagonist that became available for long-term treatment of obesity in the United Kingdom and other countries beginning in 2006 [27]. In the United States, the FDA did not approve the agent because of its association with a risk of psychiatric disorders including depression and suicidal tendencies [27]. In 2009, the EMEA (European Medicine Agency) withdrew market authorisation for rimonabant in all countries of the European Union due to safety concerns [21]. Sibutramine, a popular antiobesity drug used worldwide since 1997, was withdrawn from the European and US markets in 2010 because of safety concerns [27,28]. In the Sibutramine Cardiovascular Outcomes (SCOUT) trial, the drug was shown to be associated with increased risk of major adverse cardiovascular events (a composite of non-fatal heart attack, non-fatal stroke, resuscitation after cardiac arrest, and cardiovascular death) in obese patients, as compared to placebo [29]. 

The medications currently approved by the FDA for use in the United States include phendimetrazine, benzphetamine, diethylpropion, phentermine, and orlistat. The US Drug Enforcement Administration classifies phendimetrazine and benzphetamine as schedule 3 drugs and diethylpropion and phentermine as schedule 4 drugs [20,30]. These drugs are recommended for the short-term (12 weeks or less) treatment of obesity [20,30]. 

Orlistat is the only drug currently approved by the FDA for long-term management of obesity [21,31]. It acts by reversibly inhibiting pancreatic lipase enzyme, thus preventing the hydrolyzation and absorption of dietary fat by approximately 30%, and thereby decreasing the caloric intake of obese patients [32]. A meta-analysis of 29 studies of orlistat for weight loss in adult patients reported a weight loss of 2.59 kg and 2.89 kg at 6 months and 12 months, respectively [33,34]. In comparison to patients on diet and placebo only, the patients treated with orlistat demonstrated a significant improvement in blood glucose concentrations, insulin resistance, waist circumference, total and low-density lipoprotein cholesterol (LDL-C), and blood pressure [35-37]. Owing to the unabsorbed fat in the intestine, orlistat causes some side effects, among which the most common are diarrhea, flatulence, bloating, abdominal pain, and dyspepsia [38].

The only currently approved long-term therapy for obesity in the United States is not only having limited efficacy, but is also associated with number of side effects. Thus, a dire need exists for the development of novel antiobesity drugs with high efficacy and long-term safety. In recent years, there has been an explosion in knowledge of the mechanisms involved in the regulation of feeding behavior and the cellular mechanisms involved in energy expenditure. This has led to a rich harvest of new potential targets and drug candidates that may offer hope of safe and effective treatments for obesity.

## NOVEL TARGETS AND DRUG CANDIDATES

### Drugs that Decrease Energy Intake

These include appetite suppressants and lipase inhibitors. Among the appetite suppressants, the targets are classified according to the energy pathways they modulate. These can either be stimulators of anorexigenic pathways or inhibitors of orexigenic pathways (Fig. **4**). The lipase inhibitors inhibit dietary fat absorption and hence decrease energy intake.

### Appetite Suppressants: Stimulators of anorexigenic pathway

#### Leptin

 Leptin treatment has proved to be ineffective for attaining weight reduction in obese individuals. However, the use of low doses of leptin to maintain weight loss attained by other anorectic medicines and lifestyle modifications is emerging as a pharmacotherapeutic option [39,40]. In human beings, the maintenance of reduced body weight is achieved via coordination of metabolic, neuroendocrine, and autonomic responses that normally favor weight regain following a loss [40]. Weight loss decreases energy expenditure, reduces the tone of the sympathetic nervous system, and decreases the circulating levels of leptin and thyroid hormones [40]. The weight-reduced state is considered to be a state of relative leptin deficiency [41-43]. These counter-regulatory changes induce hyperphagia and a hypometabolic state, which result in tachyphylaxis to anorectic medications and favor weight regain. Administration of low doses of leptin in humans after weight loss has been shown to reverse these counter-regulatory neuroendocrine responses and increase energy expenditure, thus maintaining the weight reduction [40,44].

#### Leptin mimetics

 To overcome leptin resistance (Fig. **3**), drugs that can bypass the leptin transporter are being explored. These include oral leptin mimetics and intranasal leptin, both of which are in preclinical stages of drug development [15,45,46].

#### Stimulating leptin signaling

Recent preclinical evidence has shown that coadministration of amylin with leptin improves leptin-mediated STAT3 signaling in the ventromedial hypothalamus of rats with diet-induced obesity [47]. This observation suggests that combination therapy with leptin and amylin has potential for the treatment of obesity. One such combination therapy is currently being evaluated in clinical trials and is discussed later in this article.

Another strategy to improve sensitivity to leptin is to inhibit the autoinhibitory factors involved in leptin signaling, i.e., suppressor of cytokine signaling-3 and PTP1B. This strategy is particularly compelling as the activity of these autoinhibitory factors is increased in obesity, hinting at an etiological role for them in leptin resistance [15, 48,49]. Suppressor of cytokine signaling-3 haploinsufficiency in mice enhances the weight-reducing effects of leptin and confers resistance to diet-induced obesity [50]. PTP1B is a negative regulator of the leptin and insulin signaling pathways, so it has emerged as an attractive novel target for the treatment of both type 2 diabetes and obesity [51]. The development of PTP1B inhibitors for the treatment of obesity is in preclinical stages. One PTP1B inhibitor, trodusquemine, has been shown to suppress appetite, reduce body weight, and improve plasma insulin and leptin levels in a murine model of diet-induced obesity [52].

Another logical strategy to overcome leptin resistance is to manipulate leptin-regulated pathways distal to the first-order neuron, which is the melanocortin pathway.

#### Melanocortin pathway stimulation

In the leptin-melanocortin pathway, POMC is the first key intermediary downstream of leptin-receptor signaling. Pharmacological mechanisms to increase POMC expression are not evident, due to the involvement of POMC-derived peptides in adrenal physiology [53]. Hence, the downstream pathways are targeted for drug development.

#### Melanocortin receptor agonists

Cleavage of POMC produces, among other peptides, α-MSH, which activates melanocortin 3 and 4 receptors (Mc3r, Mc4r) to exert catabolic effects [54]. These receptors are highly promising targets for obesity treatment because of their vital functions and relative specificity in energy homeostasis [55]. Ample genetic evidence proves Mc4r signaling to be a critical component of the body-weight regulation system. Null mutations in Mc4r cause pronounced, dominantly inherited, monogenic obesity in rodents and humans, and are associated with increased food intake, decreased energy expenditure, and increased lean body and fat mass [56,57]. These observations have led to the development of Mc4 receptor agonists; however, their development has proceeded less successfully than expected due to the melanocortin pathway’s role in increasing heart rate and blood pressure as well as induction of penile erection [58,59]. These effects cannot be dissociated from that of appetite. There is also ample evidence to demonstrate that stimulation of the melanocortin pathway by inflammatory cytokines results in cachexia. This is another issue of concern with long-term use of melanocortin agonists [60,61]. Some Mc4 receptor agonists have been tested in preclinical and clinical studies. A melanocortin 4 receptor agonist, 3 RY764, reduced food intake and augmented erectile activity in rodents [62]. MK-0493, another potent and selective agonist of Mc4 receptor, was associated with statistically insignificant weight reductions in a clinical trial[63]. (Table **3**) Given these discouraging results, Mc4 receptor agonists are currently not being developed for the treatment of obesity. However, they are being explored for other conditions such as sexual dysfunction [64]. Melanocortin antagonists are also being evaluated for the treatment of cachexia [65,66].

#### Melanocortin receptor signaling

Recent evidence indicates the single-minded homologue 1 (SIM1) transcription factor acts as a proximal mediator for the anorectic, but not thermogenic, effects of melanocortins [67]. In rodents and in humans genetic evidence demonstrates that the loss of SIM1 causes hyperphagic obesity in addition to causing resistance to the anorectic effects of melanocortins [68,69]. Conversely, SIM1 overexpression reduces food ingestion and body weight in mice fed a high-fat diet, acting downstream of melanocortin receptors [70]. These observations identify SIM1 stimulation as a potential antiobesity strategy.

#### Ciliary neurotrophic factor

Ciliary neurotrophic factor (CNTF) is a glial cell-produced neuroprotective cytokine. It has been explored for the treatment of neurodegenerative diseases. Unexpectedly, subjects receiving CNTF in clinical trials for this indication experienced weight losses of 10--15%, prompting researchers to consider using CNTF to treat obesity [71]. CNTF either cross-reacts with leptin receptors or directly activates its own receptors present on the hypothalamus, initiating a transduction pathway analogous to that of leptin [72]. In hypothalamic feeding centers, CNTF stimulates the proliferation of neurons that contain leptin-responsive elements [73]. Based on these promising findings, axokine, a recombinant human variant of CNTF, was used for testing in humans. Modestly successful results were observed in phase 1 and 2 clinical trials [74]. However, in one year-long phase 3 trial involving 2,000 severely obese patients, disappointing results were seen in the axokine-treated group [75]. In this trial, axokine resulted in an average weight loss of 2.9 kg, as compared to an average weight loss of 1.1 kg with placebo. While the difference was considered statistically significant, it fell short of the goal set by the FDA for the approval of antiobesity drugs, which is a 5% weight loss beyond that achieved with placebo. (Table **3**) This limited efficacy was due to the development of anti-CNTF antibodies in these patients [75]. CNTF congeners that do not elicit an immune response would be rational antiobesity drug candidates in the future [15].

#### Reuptake inhibitor of serotonin, noradrenaline, and dopamine

Tesofensine increases monoaminergic transmission by inhibiting the neuronal uptake of serotonin, dopamine, and noradrenaline, thus causing appetite suppression. In phase 2b clinical trials, this drug achieved degrees of weight loss that were significantly greater than those achieved with any other currently available antiobesity drug [76]. Over a period of 6 months, patients lost an average of 12.8 kg, 11.3 kg, and 6.7 kg on the 1 mg, 0.5 mg, and 0.25 mg doses, respectively, as compared to a 2.2 kg weight loss in the placebo group [76]. The most common adverse effects in the tesofensine group were dry mouth, nausea, constipation, hard stools, diarrhea, and insomnia. Tesofensine also increased blood pressure, heart rate, and frequency of mood changes. Tesofensine at doses of 0.5 mg and 1.0 mg increased heart rate by 7.4 and 8.1 beats per min, respectively [76]. This effect on heart rate is an important safety issue that needs special attention in future trials since obese individuals are at increased cardiovascular risk. Another issue of concern that must be comprehensively explored in future trials is the association between tesofensine and increased frequency of agitation and mood changes. Its efficacy and tolerability is currently being evaluated in a phase 3 trial [77] (Table **2**).

#### Serotonin (5- hydroxytryptamine or 5HT) receptor modulation

The nonselective serotonergic antiobesity drugs fenfluramine and dexfenfluramine have been associated with severe side effects like valvular heart disease and pulmonary hypertension; as a result, these drugs were discontinued [78,79]. A detailed analysis of the antiobesity action of fenfluramine disclosed that it directly activates hypothalamic POMC neurons through 5-hydroxytryptamine 2C (5-HT2C) receptors that are expressed on a majority of these cells [80]. This finding led to selective targeting of 5-HT2C receptors in the brain. Lorcaserin is a selective agonist of the 5-HT2C serotonin receptor in the hypothalamus, which regulates satiety and the metabolic rate [81]. In a phase 2 clinical study, patients who were administered 20 mg/day lorcaserin attained an average weight loss of 3.6 kg, which was significantly greater than the average weight loss of 0.3 kg in the placebo group [82]. 

 Two phase 3 trials BLOOM (Behavioral modification and Lorcaserin for Overweight and Obesity Management) and BLOSSOM (Behavioral modification and LOrcaserin Second Study for Obesity Management) evaluated obese and overweight patients to demonstrate the efficacy of lorcaserin (10 mg twice daily) over placebo [83.84]. In the BLOOM trial, patients treated with lorcaserin achieved highly significant and absolute weight loss in 1 year, and continuous treatment with lorcaserin over 2 years helped two-thirds of the patients maintain a weight loss of 5% or greater [83]. The average amount of weight lost on lorcaserin treatment was 5.8 ± 0.2 kg , compared with 2.2 ± 0.1 kg in the placebo group (*P*<0.001). Lorcaserin was very well tolerated; it did not result in increased risk of depression or the development of cardiac valvular insufficiency. The BLOSSOM trial confirmed the results reported by the BLOOM trial [84]. In this 52-week trial, 47.2% of patients in the lorcaserin group lost at least 5% of their body weight, compared to 25% of patients in the placebo group [84]. In the lorcaserin group, 22.6% of patients lost at least 10% of their body weight, as compared to 9.7% of patients in the placebo group [84]. Lorcaserin had successfully completed phase 3 trials and its new drug application was reviewed by the FDA<B87></C. The drug failed to get FDA approval because of safety concerns Related to the finding that high doses of the drug were associated with tumor formation in rats [85] (Table **3**).

5-HT6 receptors have also been targeted for the treatment of obesity. Central application of antisense oligonucleotide against 5-HT6 receptors decreased food intake in rats [86]. Selective 5-HT6 receptor antagonists have also been shown to reduce body weight in mice with diet-induced obesity [87]. However, recent evidence suggests the distribution of 5-HT6 receptors in the mouse brain is very different from that in rats or humans [88]. As a result, the potential role of the 5-HT6 receptor in regulating human energy homeostasis requires additional evaluation.

#### Histamine H3 receptor antagonists

Histamine-synthesizing neurons in the tuberomammillary nucleus project to the paraventricular nucleus and the ventromedial hypothalamus, two hypothalamic regions recognized to regulate food intake [89]. In 2001, Masaki *et al*. reported that central infusion of histamine in a leptin-resistant mouse model of obesity reduced fat accumulation, decreased leptin, and improved insulin sensitivity [90]. These findings initiated the development of H3 receptor antagonists, which increase histamine levels in the hypothalamus and decrease food intake. Novo Nordisk extensively investigated the antiobesity action of the potent and selective non-imidazole H3 receptor antagonist NNC38-1202. However, NNC38-1202 did not progress as an antiobesity pharmaceutical option due to possible drug interactions [91]. Schering plough was evaluating another H3 receptor antagonist, SCH497079, as an antiobesity drug candidate in phase 2 trial. The data of its clinical testing are not available [92].(Table **3**) A-331440, a potent and selective non-imidazole H3 receptor antagonist developed by Abbott Laboratories, reduced weight in a dose-dependent manner in an animal model using mice with diet-induced obesity and it prevented weight gain in genetically obese (ob/ob) mice [93]. The weight loss of the mice with diet-induced obesity was associated with reductions in both food consumption and plasma leptin levels.

#### Hoodia gordonii extract

The *Hoodia gordonii *cactus plant was traditionally used by the Bushmen of the Kalahari Desert to suppress hunger. The South Africa’s Council for Scientific and Industrial Research (CSIR) investigated the appetite suppressant activity of *Hoodia* extracts and made a breakthrough with structure elucidation of an active ingredient, p57 [94]. In 1998, CSIR licensed an agreement with Phytopharm (a British pharmaceutical company specializing in Phytomedicines) to further develop p57 [94]. Phytopharm collaborated with Pfizer for development and commercialization of the extract In an early clinical trial of 19 obese subjects, either P57 or placebo was administered for 15 days. A statistically significant reduction was observed in the average daily calorie intake in the P57 group compared with the placebo group, and no serious adverse effects were experienced by any of the subjects [94,95]. However, in 2003, Pfizer ended its collaboration with Phytopharm on the further development of p57 citing difficulties in synthesizing the extract into drug form [94,96] (Table **3**). In 2004, Unilever entered into an agreement with Phytopharm to start marketing *Hoodia gordonii *commercially in the form of a functional food for obesity management [94]. However, in 2008, Unilever severed its agreement with Phytopharm as the extract could not live to their expectations of safety and efficacy [94]. (Table **3**) Much work remains to be done before the scientific evidence supports the use of Hoodia plant extract as an appetite suppressant.

#### Nesfatin

Nesfatin-1/nucleobindin 2 (NuCB2) is a naturally occurring satiety molecule produced in the brains of mammals [97]. Chronic intracerebroventricular injection of nesfatin-1 in rats has been shown to reduce body weight, whereas that of antisense oligonucleotide against the NUCB2-encoding gene increased body weight [98]. These findings suggest nesfatin and its analogues have potential as possible antiobesity drug candidates.

#### Corticotrophin releasing factor (CRF)/urocortin

The CRF-like neuropeptide (urocortin), acting via CRF2 receptors in the brain, is a potent suppressor of appetite [99]. Administration of urocortin-3 in the ventromedial hypothalamus suppresses feeding and elevates blood glucose levels in rats, suggesting the activation of CRF2 receptor in the brain promotes stress-like responses [100].It was recently shown that transgenic mice overexpressing urocortin-3 were protected against the untoward metabolic consequences of an obesogenic high-fat diet challenge [101].These observations indicate urocortin may be another antiobesity drug target with promise.

#### Neuromedin U

Neuromedin U (or NmU) is a neuropeptide found in the brains of humans and other mammals. This peptide is involved in diverse physiological functions, including appetite suppression [102]. Intracerebroventricular administration of neuromedin U in rats is associated with marked reductions in body weight [103]. On the other hand, injection of an antibody to neuromedin U increased food ingestion [103]. In mice with diet-induced obesity, intracerebroventricular infusion of NmU for 7 days decreased body weight and total energy intake as compared with the mice given intracerebroventricular infusion of vehicle [104]. The role of neuromedin U as an appetite suppressant acting through NmU receptor 2 (Nmur2) has recently been identified from the studies using Nmur2 -/- mice (carrying 2 copies of the mutant Nmur2 allele) [104,105]. These mice were resistant to central NmU induced weight loss when fed a high fat diet as compared to wild type mice [105]. 

#### Fatty acid synthase (FAS) inhibitor

FAS enzyme is involved in the conversion of malonyl CoA to palmitate. Inhibition of FAS by a specific FAS inhibitor, C75, has been shown to reduce food intake and body weight in mice [106]. This anorectic effect is presumably mediated via interference with the expression of orexigenic neuropeptides in the brain. FAS inhibitors also stimulate carnitine palmitoyl transferase 1 (CPT-1), the rate-limiting enzyme for mitochondrial fatty acid oxidation [107].Thus, FAS inhibitors also show potential as antiobesity agents.

#### Bombesin and bombesin-like peptides

 Bombesin (and its family of bombesin-like peptides) modulates various biological and behavioral functions in humans. Intensive psychopharmacological studies in animals and humans have shown it to be an appetite suppressant [108]. Lieverse *et al*. demostarted that intravenous infusion of bombesin reduced the intake of a carbohydrate- rich meal in human subjects [109-111]. However, the satiety inducing effects of bombesin were observed in lean subjects, and not in obese individuals [111]. 

BRS-3 (bombesin receptor subtype 3) ligands comprise a new class of potential antiobesity agents that may strongly suppress feeding [112]. In animal studies, the selective antagonist of BRS3 increased food intake and body weight, whereas the selective BRS3 agonists increased metabolic rate and reduced food intake and body weight. Prolonged high levels of receptor occupancy by BRS3 agonists continued to cause weight loss in animal studies, suggesting a lack of tachyphylaxis [113].

#### Gastrointestinal peptides

 In response to ingested nutrients, a number of peptides are released from the gastrointestinal tract. These include cholecystokinin (CKK), glucagon-like peptide 1(GLP-1), peptide YY (PYY), pancreatic polypeptide (PP), oxyntomodulin, amylin, and enterostatin. These peptides act in concert with other postprandial gastrointestinal signals (e.g., gastric distention) to cause satiation and promote meal termination [7-10]. Thus, stimulation of each is being researched to determine their potential as antiobesity targets.

#### Cholecystokinin receptor agonists

CCK decreases food intake in numerous species including humans. It has been proposed to act as a satiety signal via CCK1 receptor activation [114]. A few non-peptide, selective CCK1 receptor agonists (GI-181771X [GlaxoSmithKline], CE326597 [Pfizer]) had been evaluated in clinical trials for obesity [115]. However, none of these compounds are currently undergoing clinical development. GI181771X, when given to obese patients for 24 weeks, failed to produce any reduction in body weight or waist circumference or to alter cardiometabolic risk markers [116]. (Table **3**) A possible explanation for the failure of CCK1 agonist monotherapy might be the development of tachyphylaxis upon chronic stimulation of the receptor [117]. Though CCK monotherapy has not proven successful, some studies have reported an enhanced effect of leptin on weight reduction when it is co-administrated with CCK in rats [118]. Future studies should focus on co-administration of CCK1 receptor agonists along with leptin or other hormones for the treatment of human obesity [9]

#### Glucagon-like peptide 1

The protease-resistant GLP-1 (glucagon-like peptide 1) congener, exenatide, is already marketed for the treatment of diabetes. In clinical trials, exenatide has been shown to reduce hemoglobin A1C, while also inducing modest but progressive weight loss that persists for at least 2 years [119]. Exenatide given subcutaneously twice daily at doses of 5 or 10 µg elicited mean weight reductions of up to 2.8 kg at 30 weeks and 5.3 kg at 3.5 years [120-122]. Exenatide administered at doses of 0.8 mg or 2 mg once weekly resulted in mean weight reductions of up to 3.8 kg at 15 weeks and 3.7 kg at 30 weeks [123,124]. Liraglutide is a novel GLP1 agonist that was approved by the FDA in January 2010 for the treatment of type 2 diabetes [125]. It has a long duration of action, is suitable for once-daily dosing and is more homologous to native GLP1 than exenatide [126]. Liraglutide has also shown promising results in clinical trials for the treatment of obesity in individuals without diabetes, resulting in weight losses of 4.8--7.2 kg with different doses ranging from 1.2 to 3 mg [127]. This was significantly higher than that seen with placebo (*P* = 0.003 for 1.2 mg liraglutide and *P* < 0.0001 for 1.8--3 mg liraglutide) and orlistat (*P* = 0.003 for 2.4 mg liraglutide and *P* < 0.0001 for 3 mg liraglutide). More than 50% of the patients on liraglutide therapy achieved a weight reduction in the range of 5--10% [127]. Nausea and vomiting were the most common side effects. These were transient in nature and rarely led to the discontinuation of therapy. Liraglutide has progressed to phase 3 trials for the treatment of obesity [128] (Table **2**).

#### Peptide YY3-36 and pancreatic polypeptide

Peripheral administration of peptide YY 3-36 (PYY3-36) inhibits food ingestion and reduces body weight in rodents, apparently by activating autoinhibitory Y2 receptors on orexigenic NPY/Agrp neurons in the hypothalamus, thereby derepressing adjacent anorexigenic POMC neurons [129]. In obese individuals, PYY3-36 intravenous infusion has been reported to ease hunger, decrease single-meal food intake by 30%, and significantly reduce the cumulative 24-hour caloric intake [130]. This treatment was well tolerated, with no complaints of altered food palatability or nausea. Based on these promising results, an intranasal formulation of PYY3-36 was evaluated in clinical trials [131]. However, this formulation demonstrated disappointing results; the amounts of weight lost at low doses were similar to those lost with placebo. Weight loss at high doses of intranasal PYY could not be assessed because 60% of patients dropped out due to nausea and vomiting. This high incidence of nausea with intranasal administration might be related to PYY’s C-max (peak serum concentration of drug), since concentration spikes with the nasal route of delivery [132]. Novel formulations providing sustained levels of PYY might be well tolerated and are desirable targets for future research. 

PP is another gut-derived peptide with similar action to PYY. Obinepitide, a synthetic analogue of both PYY3-36 and PP and an agonist of Y2 and Y4 receptors, is under clinical development [128]. (Table 2) In obese human subjects, once-daily subcutaneous dosing of obinepitide significantly inhibited food intake up to 9 hours after dosing [128]. 

#### Oxyntomodulin

Oxyntomodulin is a product of the proglucagon gene from which GLP-1 is cleaved. In humans, intravenous infusions of oxyntomodulin have been shown to acutely lessen hunger and single-meal food intake without reducing food palatability [133]. Repeated subcutaneous injections of oxyntomodulin for 4 weeks reduced body weight by up to 2.3 ± 0.4 kg as compared with 0.5 ± 0.5 kg in the control group (*P* = 0.0106) [134]. Importantly, oxyntomodulin reduced single-meal intake by 25% at the start of this study and by up to 35% at the end, indicating there was no tachyphylaxis related to its anorectic effects [134]. In another study, subcutaneous injection of oxyntomodulin for 4 days reduced energy intake by 17.3 ± 5.5%, with no change in meal palatability [135]. Oxyntomodulin did not alter resting energy expenditure, but it increased activity-related energy expenditure by 26.2 ± 9.9% and total energy expenditure by 9.4 ± 4.8%. Reductions in body weight of 0.5 ± 0.2% were also observed during the period of oxyntomodulin administration. These modest and favorable results led to the development of the potent and synthetic long-acting oxyntomodulin analogue, OAP189 (TKS1225), which is currently being investigated in a phase 1 clinical trial [136,137] (Table **2**).

#### Amylin

 Amylin is a peptide that is postprandially cosecreted with insulin from pancreatic cells. Food intake causes a rapid increase in plasma amylin that is directly proportional to the meal size [138,139]. Amylin functions as a satiety signal by acting on area prostema in the hindbrain [140]. Administration of exogenous amylin prior to a meal dose-dependently decreases meal size in rodents thereby reduces food intake [141,142]. Amylin also suppresses gastric emptying, gastric acid output, and glucagon secretion. Pramlintide, a synthetic amylin analog, is currently marketed for the treatment of diabetes, but it has also been found to induce mild progressive weight loss in humans for at least 16 weeks [143]. In one study, subjects completing treatment with pramlintide experienced placebo-corrected reductions in body weight of 3.7 ± 0.5% [144]. Approximately 31% of the pramlintide-treated subjects achieved a weight loss of 5% or greater, as compared with 2% in the placebo group [144]. To date, much of the research with amylin has focused on its potential to increase leptin sensitivity. Infusions of an ineffective dose of leptin (500 µg/kg/day had no significant effect on the body weight of rodents with diet-induced obesity) in combination with a moderately effective dose of amylin (100 g/kg/day achieved weight losses of up to 6.5%) have been shown to elicit marked, synergistic weight loss (up to 12%) in rats with diet-induced obesity [47]. These findings provide a rationale for using pramlintide in combination with leptin for the treatment of obesity. This combination is currently being evaluated in clinical trials and is discussed again later in this article.

#### Enterostatin

Enterostatins are pentapeptides generated in the small intestine as a result of N-terminal proteolytic processing of pancreatic procolipase. Enterostatin is absorbed from the gut and acts as a powerful anorectic peptide, particularly in regards to reducing the intake of fatty foods [145]. In a study performed in rats, chronic intracerebroventricular injections of enterostatin decreased intake of a high-fat diet and reduced body weight [146]. Previous experiments explored the hypothesis that an association exists between human obesity and enterostatin dysregulation [147,148]. However, in clinical trials, intravenous enterostatin failed to show any effect on feelings of hunger, satiety, or food preference [149]. Present knowledge suggests the molecular identification of enterostatin receptors is required in order for enterostatin to be developed further as antiobesity drug target.

### Appetite Suppressants: Inhibitors of Orexigenic Pathway

#### Neuropeptide Y receptor antagonists

 Neuropeptide Y (NPY) is a 36 amino-acid peptide that was found to potently stimulate food intake in a variety of species [10]. This led to a surge of interest in developing NPY receptor antagonists as antiobesity drugs. The orexigenic effects of NPY are chiefly mediated by the Y1 and Y5 receptors. Thus, antagonists for these receptors were viewed as potential antiobesity drugs. Neuropeptide Y1 antagonists demonstrated promising weight-reducing results in animal studies, whereas neuropeptide Y5 antagonists showed equivocal effects [150-154]. Few of these antagonists progressed to clinical trials, and since they did not live up to expectations, none are being currently pursued. NGD 95/1, a NPY1 antagonist, was evaluated in a phase 1b clinical trial conducted by Neurogen and Pfizer, but it was not developed further because of elevated liver enzymes [10]. MK-0557, an orally active NPY5 receptor antagonist, was evaluated in a year-long clinical trial, but since it did not produce clinically meaningful weight loss, further development was abandoned [155] (Table **3**).

In addition to the disappointing clinical experience with NPY antagonists, the critical role of NPY in energy regulation was also disputed by the finding that NPY deficiency did not result in leanness in mice [15,156]. However, NPY deficiency in ob/ob (leptin deficient) mice was found to attenuate the obesity phenotype by reducing food intake and increasing energy expenditure [157]. This observation implies that NPY is required for a full response of leptin deficiency, even though it might not be required to maintain body weight. Thus, its blockade might be useful for preventing the regain of weight lost by other means [15]. More recently, interest has focused on the stimulation of presynaptic NPY2 receptors in the arcuate nucleus as a mechanism to reduce NPY release and, thus, food intake [131]. As discussed above, peptide YY (PYY), a gut hormone peptide with selectivity for NPY2 receptors, has been explored in clinical trials.

#### Melanin concentrating hormone 1 receptor antagonist

 Melanin-concentrating hormone (MCH) is a 19-amino acid, cyclic neuropeptide that is present in the hypothalamus. MCH administration or its transgenic overexpression in mice increases body weight by stimulating food ingestion and adipogenesis, while decreasing energy expenditure [158,159]. This suggests that MCH1 receptor antagonists might promote weight loss, but there are several issues of concern with the development of these compounds. MCH1 receptor knockout mice demonstrate weight reduction, hyperactivity, and increased energy expenditure, but they are also hyperphagic [160]. This situation of chronically elevated energy intake due to marked hyperactivity and energy expenditure might be hazardous, as ample evidence suggests that long-term caloric restriction increases lifespan, and chronic increased calorie intake in the face of increased energy expenditure can theoretically shorten life span [161-163]. MCH antagonism also poses a risk of cardiac arrhythmias, and MCH is known to modulate many functions beyond feeding, such as locomotor activity, anxiety, aggression, sensory processing, learning, and sleep cycle [159,164,165]. Thus, it might be challenging to design anti-MCH agents that selectively regulate energy homeostasis without exerting adverse side effects. Early clinical testing of the MCH1 receptor antagonist NGD-4715 failed after recipients complained of vivid dreams and awakenings [166].

#### Orexin antagonists

 Orexin A and orexin B (or hypocretin-1 and -2) are a pair of excitatory neuropeptides present in the brain. These peptides have diverse physiological functions like the regulation of feeding, the sleep-wake cycle, and the reward pathway [167,168]. It has been reported that intracerebroventricular injection of orexin increases food intake in rats [10,167,168]. The antiobesity activity of a prototype selective orexin-1 receptor antagonist, SB-334867-A, has been explored in preclinical studies [169]. This drug reduced cumulative food intake and body weight in genetically obese mice when administered over a period of 14 days [169]. Stimulation of thermogenesis also occurred, as evidenced by an increase in uncoupling protein-1 mRNA expression in intrascapular brown adipose tissue. A single dose of SB-334867-A was found to increase metabolic rate over a period of 4 h in ob/ob mice [170].

#### Galanin antagonists

 Galanin, a 29-30 amino acid neuropeptide, is widely distributed in both the central and peripheral nervous systems. Pharmacological studies indicate galanin plays a role in regulating feeding and nutritional balance [10,171].Injection of galanin into the central nucleus of the amygdala and the paraventricular nucleus of the hypothalamus in rats significantly increases food intake [172]. Central injection of galanin stimulates the intake of fatty foods in particular [173].Similarly, galanin gene knock-out mice also demonstrate significantly lower intake of fat in comparison with wild-type mice [174]. However, repeated galanin infusions in the third ventricle in rats failed to induce obesity or hyperphagia, suggesting tachyphylaxis may have developed [175]. Thus, further evaluation of the role of galanin in modulating feeding behavior is required. To date, three galanin receptors have been cloned and attempts are underway to synthesize specific galanin antagonists for the treatment of obesity [10,176].

#### Ghrelin antagonist

 Ghrelin is an orexigenic hormone secreted from the stomach and upper intestine. Its plasma levels rise before the start of a meal (coincident with meal times of a fixed feeding schedule) and fall again rapidly after the meal is initiated [177]. This pattern of secretion implies that ghrelin plays a role in mealtime hunger and meal initiation and has led to the development of ghrelin antagonists for the treatment of obesity. However, the role of ghrelin antagonism in this aspect has been challenged by studies performed with ghrelin-deficient mice. Mice with congenital deletions of the gene encoding ghrelin or its receptor exhibited minimal body weight reductions on standard chow but they were resistant to highfat-diet-induced obesity [178,179]. In spite of these observations, a number of other studies demonstrated that ghrelin antagonism in adult animals reduces body weight [180,181]. Ghrelin receptor antagonists have been found to decrease energy intake in lean mice, obese (ob/ob) mice, and mice with diet-induced obesity [182]. They also reduce the rate of gastric emptying. Repeated administration of ghrelin receptor antagonist has been shown to decrease body weight gain and improve glycemic control in ob/ob obese mice [183]. The possible efficacy of ghrelin inhibitors as weight-loss agents is consequently being reevaluated. Evidence is also emerging that suggests there are chronic effects of ghrelin on fat accumulation by modulation of hypothalamic genes coding for the peptides and receptors involved in energy balance regulation, independent of food intake [184].

### Lipase Inhibitors

Lipase inhibitors inhibit gastric and pancreatic lipases in the lumen of the alimentary tract to decrease systemic absorption of dietary fat, without affecting food intake. The lipase inhibitor presently in clinical development for the treatment of obesity is cetilistat [185] (Table **2**) The clinical data currently available suggest that cetilistat has comparable efficacy to orlistat (Phase 2b clinical trial showed 3 kg weight loss over placebo in 3 months). Also, as compared to orlistat, this drug has a favorable side-effect profile (90% fewer severe gastrointestinal side effects) [186,187].

## DRUGS THAT INCREASE ENERGY EXPENDITURE AND INCREASE METABOLIC ACTIVITY, THERMOGENESIS, AND LIPOLYSIS PERIPHERALLY

### Thyroid Hormone Receptor Agonist

Thyroid hormones are known to stimulate weight loss by increasing the metabolic rate. However, their use has been associated with cardiac stimulation and protein loss. Recently, several subtypes of the thyroid receptors have been discovered. The alpha subtype is located in the heart and mediates the cardiostimulatory effect of the thyroid hormones. The beta subtype is chiefly located in the liver and is involved in hepatic cholesterol metabolism. A beta subtype-specific agonist, KB-141, has been developed [188]. In one study, KB-141 was administered orally to obese zucker fa/fa rats for 21 days and ob/ob mice for 7 days, after which body weight, adiposity, and lipid levels were examined [189]. In fa/fa rats, KB-141 reduced body weight by 6--8% and adiposity by 5--6%, without causing tachycardia. In ob/ob mice, KB-141 lowered serum cholesterol, triacylglycerols, and both serum and hepatic levels of free fatty acids, without causing tachycardia. Treatment of ob/ob mice with KB-141 for 2 weeks improved glucose tolerance and insulin sensitivity in a dose-dependent manner with no effect on heart rate [189]. Another thyroid beta subtype receptor agonist, GC1, when administered to cynomolgus monkeys for 7 days, caused significant reductions in cholesterol and lipoprotein (a), and an average weight loss of 4%, without causing cardiac stimulation [190].

### Human Growth Hormone Analogue

Growth hormone has profound lipolytic and antilipogenic activity and is known to reduce body fat and cause weight loss. AOD-9604 is an orally active analogue of peptide fragment of human growth hormone (hGH 177--191) that selectively activates lipolysis in adipose tissue. In one study, AOD-9604 reduced body weight in genetically obese zucker rats and ob/ob mice, without inducing the untoward effect on glycemic control normally observed with growth hormone [191]. In a 12-week randomized clinical trial, subjects receiving AOD-9604 (1 mg/d) lost an average of 2.6 kg, compared to 0.8 kg in the placebo group [192]. Development of this drug was terminated in 2007 as the drug failed to induce significant weight loss in a 24-week trial of 536 subjects [192] (Table **3**).

### β_3_-adrenoceptor Agonists

β_3_-adrenoceptor agonists were first described as an antiobesity target in 1983, and their seven-transmembrane G-protein-coupled β_3_-adrenoceptor was cloned 6 years later [193,194]. To date, however, β_3_-adrenoceptor agonists have not been marketed for the treatment of obesity. The first generation β_3_-agonists were selected on the basis of their thermogenic and antiobesity actions observed in rats and mice. In obese rodents, they produced good weight loss results by increasing metabolic rate. Unfortunately, clinical experience with these compounds has been less encouraging. The side effects of first-generation compounds in humans were tachycardia and tremors, indicating lack of β_3_ receptor selectivity. They were also not effective at reducing weight by increasing thermogenesis, since their agonistic potential for human β_3_ receptors was not as strong as for rodent β_3_-adrenoceptors [10,195,196]. In rodents, the β_3_-receptors are present in both white adipose tissue and brown adipose tissue (BAT), but in humans, β_3_ receptors are predominantly present in BAT. Newborn infants have relatively large amounts of BAT, which plays a thermoregulatory role in affording protection from cold exposure. However, with increasing age, the amount of BAT decreases. Thus, it has been argued that the expression of β_3 _receptors might be insufficient in human adults for β_3_-adrenoceptor agonists to elicit a response [10,195]. Treatment of obese patients with the first-generation β_3 _agonists BRL26830 and BRL35135 had no effect on thermogenesis or fuel metabolism. These drugs also caused undesirable tremors and tachycardia [195, 197, 198].

The presence of BAT and its physiological role in adults has been a subject of great debate. Recently, the presence of BAT in adult humans was demonstrated with radiological evidence [199]. In addition, body mass index and the amount of BAT have been shown to be inversely proportional, especially in older patients; this suggests BAT may play a protective role against obesity in adults [199]. A few studies have also shown that chronic stimulation with selective agonists causes upregulation of β_3_-adrenoceptors and induces BAT hypertrophy in rodents, dogs, and monkeys, a finding that suggests recruitment of BAT could also occur in humans. These recent developments have led to a resurgence of research into drugs targeting β_3 _receptors. Novel generation of highly selective β_3 _receptor agonists have been tried in clinical trials [10, 19, 200, 201]. CL 316243 increased insulin-mediated glucose disposal and fat oxidation in human volunteers, without causing tremors and cardiac stimulation [200]. However, the poor oral bioavailability of CL316243 is a major drawback. Prodrugs with improved bioavailability have also been developed [201]. The oral bioavailability of a single dose of LY377604 was greater than 20%, and in obese sbjects it was shown to increase metabolic rate by 17.5% [10]. Another β3 agonist N5984, developed by Nisshin Kyorin Pharmaceuticals was in early phase of clinical testing. However, the clinical study data for N5984 is not available [202] (Table **3**).

### Uncoupling Proteins

**Fig. (5).**
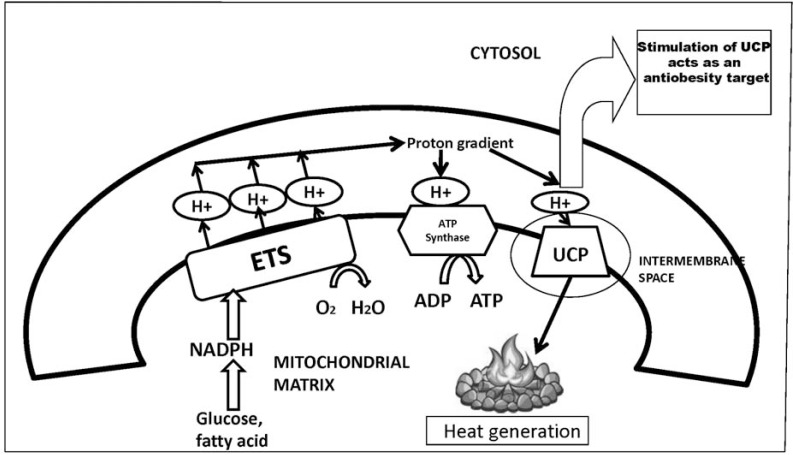
Mechanism of uncoupling proteins. UCP= Uncoupling protein UCP1: Unique to brown fat UCP2: Widely distributed in the body UCP3: Mainly in skeletal muscle Stimulation of UCP3 might have beneficial effects for obesity management ETS= Electron transport chain; ADP= Adenosine diphosphate; ATP= Adenosine-5'-triphosphate; NADPH= Nicotinamide adenine dinucleotide phosphate H^+^

Uncoupling protein 1 (UCP1) or thermogenin, a member of the mitochondrial transporter superfamily, is involved in energy expenditure and brown fat thermogenesis. UCP1 protein dissociates the mitochondrial fatty acid oxidation from production of ATP and the energy is dissipated as heat (Fig. **5**) [10, 203]. This is a key process for non- shivering thermogenesis and body weight regulation in mammals [203]. Although UCP1 is very well characterized, it has not been pursued as a target for increasing energy expenditure because the protein is uniquely present in BAT, which is barely detected in adult humans. In the past few years, a few proteins with high sequence homology to UCP1 have been discovered that are also expressed in non-BAT. These include UCP2, which is widely distributed in the body, and UCP3, which is expressed mainly in skeletal muscle [204]. Owing to the ubiquitous localization of UCP2, the likelihood of unwanted side effects makes this protein a less appealing target for obesity treatment. In contrast, stimulation of the skeletal-muscle-specific UCP3 activity could provide a more dependable mechanism by which whole-body thermogenesis in humans could be increased. Mutations in the gene encoding UCP3 have been discovered in some individuals suffering from severe obesity and non-insulin-dependent diabetes mellitus [205]. Transgenic mice with muscle-directed overexpression of UCP3 were hyperphagic and lean when fed a palatable diet, and their glucose tolerance was improved [206]. Thus, the pharmacological stimulation of UCP3 activity could result in beneficial effects against obesity and type 2 diabetes mellitus [206].

### Diacylglycerol Acyltransferase Inhibitors

Diacylglycerol acyltransferase (DGAT) enzyme catalyzes the final reaction of triacylgycerol synthesis. Two isozymes of DGAT, DGAT1 and DGAT2, have been described. DGAT1 plays an important role in the synthesis of very-low-density lipoprotein (VLDL). Since increased plasma VLDL concentrations might promote obesity, the inhibition of DGAT1enzyme is considered a possible therapeutic target for obesity treatment [207]. DGAT gene knockout mice are resistant to diet-induced obesity and appear to compensate for the reduced ability to store fat by increasing energy expenditure [208]. A potent and highly selective DGAT1 inhibitor, compound 4a, reportedly reduced weight and liver triglycerides when dosed chronically in diet-induced obese mice [209]. It also depleted serum triglycerides in a dose-dependent manner following a lipid challenge in a murine model of obesity, thus reproducing major phenotypical characteristics of DGAT1 knockout mice [209].

### Stearoyl-CoA Desaturase1 Inhibitors

Stearoyl-CoA desaturase (SCD) is the rate-limiting enzyme involved in monounsaturated fatty acid synthesis. It has recently been reported to be a vital control point regulating hepatic lipogenesis and lipid oxidation. SCD1-deficient mice exhibit increased energy expenditure, reduced body adiposity, and increased insulin sensitivity. These mice are resistant to diet-induced obesity [210]. Much evidence suggests that the direct antiobesity effect of SCD1 deficiency results from increased fatty acid oxidation and decreased lipid synthesis. All of these findings suggest that the pharmacological manipulation of SCD activity might be of benefit in the treatment of obesity, diabetes, and metabolic syndrome [211].

### Acetyl-CoA Carboxylase Inhibitors

The acetyl-coenzyme A carboxylase (ACC) enzyme plays an important role in fatty acid metabolism in most living organisms. ACC is a biotin-dependent enzyme that catalyzes the carboxylation of acetyl-CoA to produce malonyl-CoA. Mice lacking ACC2 have continuous fatty acid oxidation and reduced body fat and body weight. This validates efforts to develop an anti-obesity drug based on antagonism of this enzyme [212].

### Adipocyte-complement-related Protein or Adiponectin

Adiponectin (adipocyte-complement-related protein of 30 kDa) is a hormone produced by fat cells that is associated with fatty acid oxidation, increased insulin sensitivity, and antiatherogenic properties [213]. Adiponectin levels are inversely correlated with obesity and obesity-associated complications such as type 2 diabetes mellitus, cardiovascular disease, and hepatic dysfunction [213-216]. The association of low adiponectin levels with obesity underscores its important role in obesity-related comorbidities. It has been recognized as a key molecule in obesity as well as in the metabolic syndrome, and is a potentially promising target for the prevention and treatment of the metabolic syndrome [213, 217,218]. Famoxin is a proteolytic cleavage product of this protein. Administration of famoxin to mice has been shown to increase fatty-acid oxidation and induce weight-loss without affecting food ingestion [219]. The Genset Corporation was preparing famoxin for phase 1 clinical studies; however, further clinical development of this compound is not known when Genset was acquired by Serono Company in 2003 [220] (Table **3**). A few drugs have been reported to stimulate the secretion or to induce the expression of adiponectin, including pioglitazone, endocannabinoid (CB1) receptor antagonists, and inhibitors of the renin-angiotensin pathway [221-223].

### 11 Beta Hydroxysteroid Dehydrogenase Type 1 Inhibitors

There is strong evidence from rodent obesity models indicating that the glucocorticoid action underlies the pathology of metabolic syndrome. In these models, it was observed that removing glucocorticoids reverses obesity and its metabolic abnormalities [224,225]. 11 beta-hydroxysteroid dehydrogenase type 1 (11β-HSD1) is an enzyme that regenerates the active glucocorticoid cortisol from its inactive metabolite cortisone. Animals with 11β-HSD1 knockout have normal plasma glucocorticoid levels, but they cannot regenerate glucocorticoid inside liver and adipose tissue. As a result, they are protected from insulin resistance, hyperglycemia, and weight gain induced by high-fat feeding [225]. Conversely, mice with selective overexpression of 11β-HSD1 in adipose tissue have increased intra-adipose glucocorticoid concentrations, despite no change in plasma levels. These animals display a dramatic phenotype of central obesity, insulin resistance, and hyperglycemia [225]. In idiopathic obesity in humans, 11β-HSD1 activity in adipose tissue is selectively increased to a similar degree as that seen in transgenic 11β-HSD1 overexpressing mice [226]. Pharmacological inhibition of 11β-HSD1 to lower intracellular cortisol concentrations in liver and adipose tissue, without altering circulating cortisol concentrations or responses to stress, is an exciting potential therapy for obesity and diabetes. A few 11β-HSD1 inhibitors have been tested in preclinical studies. BVT.2733, an11β-HSD1inhibitor, has been reported to reduce food intake and weight as well as improve glucose tolerance in a mouse model of obesity and diabetes [227].

## MODULATION OF FAT STORAGE OR ALTERATION OF ADIPOCYTE DIFFERENTIATION

Peroxisome proliferator-activated receptor gamma (PPAR-γ) ligands have been reported to stimulate UCP1 expression in BAT but not in white adipocytes. This finding led to the identification of BAT-specific transcription cofactor, PPAR-γ coactivator-1 (PGC-1)[8, 228, 229]. PGC-1 is a family of transcription coactivators that functionally interact with nuclear receptor PPAR-γ [230]. It plays a cardinal role in the transcriptional programme of adaptive thermogenesis occurring in BAT as mitochondrial biogenesis, respiration, and thermogenesis [230]. Ectopic expression of PGC-1 in white adipocytes converts them to a more BAT-like phenotype [8]. The treatment of obese mice with rosiglitazone has been shown to boost PGC-1 transcription in white adipose tissue, concomitant with increased mitochondrial function and insulin sensitivity [231]. Increasing PGC-1 levels in skeletal muscle and white adipose tissue could, therefore, be potentially beneficial for the treatment of obesity and type 2 diabetes.

## MIMICKING CALORIC RESTRICTION

Caloric restriction triggers an adaptive drop in metabolic rate in rodents, nonhuman primates, and humans. This adaptation plays a central role in the rebound of body weight after weight loss observed in most patients<B19></C>; it also evokes beneficial effects in lowering the incidence of metabolic disorders related with aging, overweight or obesity, insulin resistance, and cardiovascular disease [19]. Caloric restriction has been demonstrated to increase lifespan in organisms, ranging from yeast to rodents, as well as in nonhuman primates [161-163,232,233]. Recently, a class of proteins known as SIRTS or sirtuins (also known as silent information regulator 2 [Sir2]-related enzymes) has been implicated in an effect of CR on lifespan extension and the prevention of comorbidities associated with obesity [233, 234]. Sirtuins are the family of enzymes with nicotinamide adenine dinucleotide-(NAD) dependent histone deacetylase or ADP-ribosyl transferase activity [235, 236]. The mammalian sirtuins (Sirt1--Sirt7) participate in a number of cellular and physiological functions such as gene silencing, apoptosis, mitochondrial function, energy homeostasis, and longevity [237].Transgenic mice with overexpression of the *sirt1* gene demonstrate decreased lipid-induced inflammation, improved glucose tolerance, and protection from hepatic steatosis when fed a high-fat diet [238].

## COMBINATION THERAPIES

Various fixed-dose combination antiobesity drugs are in different phases of clinical development. These include the following:

### Bupropion and Naltrexone (Contrave) 

Contrave is a fixed dose combination of bupropion and naltrexone in a single tablet. Bupropion, a nonselective dopamine- and norepinephrine-reuptake inhibitor, is considered to reduce weight by stimulating hypothalamic POMC neurons. The stimulation of hypothalamic POMC neurons is inhibited by β-endorphin as an opioid reward process is involved in the short-term control of eating [239]. In combination with bupropion, naltrexone, a µ-opioid receptor antagonist, acts in a synergistic manner to stimulate weight loss by blocking the β-endorphin-mediated inhibition of the POMC neuron [192]. In phase 3 trials, after 56 weeks of treatment, 62% of patients receiving contrave 32 (bupropion SR 360 mg/naltrexone SR 32 mg) lost at least 5% of their body weight as compared to 23% of patients in the placebo group [240]. Approximately 34% patients lost 10% or more of their body weight and 17% patients lost at least 15% of their mean body weight in the contrave group. Patients receiving contrave32 had a mean weight loss of 8 kg, as compared to 1.8 kg in the placebo group, after 56 weeks of treatment [240]. Obese patients on contrave also demonstrated significant improvements in important markers of cardiometabolic risk, including waist circumference, high-density lipoprotein cholesterol, insulin resistance and triglycerides. Contrave had completed phase 3 trials successfully [240-242]. Its NDA has recently been reviewed by the FDA. The FDA panel has deferred the approval of the drug, pending the conduction of a long-term study demonstrating cardiovascular safety [243] (Table **3**).

### Bupropion and Zonisamide (Empatic)

Empatic is a fixed-dose combination drug containing bupropion and zonisamide. Zonisamide is an antiepileptic drug that has serotonergic and dopaminergic activity in addition to its ability to act as a blockade of sodium and calcium channels. Weight loss was an adverse effect associated with zonisamide treatment in clinical trials for epilepsy. Although the exact mechanism for this weight-reducing effect is unknown, the proposed mechanism includes alterated perception of taste due to carbonic anhydrase activity and modulation of dopamine and serotonin levels in the brain [241, 244, 245].Empatic has shown promising results in phase 2 clinical trials [241] (Table **2**). Phase 2 trials demonstrated that patients completing 24 weeks of empatic360 (bupropion SR 360 mg/zonisamide SR 360 mg) and empatic-120 (bupropion 360 mg/zonisamide 120 mg) therapy attained a significant weight loss as compared to placebo (9.9%, 7.7% vs. 1.7%, respectively (p<0.001)). In the empatic-360 group, 82.6% of patients lost at least 5% of their baseline body weight and 47.7% lost at least 10% of their baseline body weight as compared to 18.9% and 5.7% patients in the placebo group, respectively [192].

### Phenteramine and Topiramate (Qnexa) 

Qnexa is a combination drug that consists of low doses of phentermine and topiramate. Phentermine is a popular appetite suppressant used for weight loss. Topiramate is approved as an anti-epileptic and antimigraine drug having multifactorial effects on the central nervous system. It is thought to act as a γ-aminobutyric acid agonist that increases satiety, although the precise mechanism of action is unclear. Qnexa has shown promising clinical data in a phase 3 trial [246]. An average weight loss of -8.1 kg and -10.2 kg was attained at the end of 56 weeks with phentermine 7·5 mg plus topiramate 46·0 mg, and phentermine 15·0 mg plus topiramate 92·0 mg, respectively. Also, there were significant improvements in cardiovascular and metabolic risk factors among patients treated with Qnexa [246]. An FDA panel recently voted against the NDA of Qnexa because of safety concerns (Table **3**). The potential benefits of the drug were overridden by its adverse effect profile, which includes its teratogenic potential, ability to increase heart rate, and propensity to cause psychiatric problems [247]. 

### Pramlintide and Metreleptin

This combination targets the long-term adiposity signals (e.g., leptin) and short-term satiety signals (e.g., amylin). This is a novel integrated neurohormonal strategy recently employed for obesity drug development. Various preclinical and clinical studies have demonstrated a synergistic effect of amylin and leptin combination in reducing food intake and body weight [47]. Amylin has also been implicated in enhancing leptin signaling [47]. The combination of the amylin analogue pramlintide and the recombinant human leptin metreleptin caused significant weight reductions (^-^11.5±kg) in comparison with monotherapy in a 24-week phase 2 clinical trial [248]. The pramlintide/ metreleptin combination was well tolerated. The most common side effects were mild-to-moderate adverse events at the injection site and nausea. This combination therapy had completed phase 2 trials successfully. However, the further clinical development of pramlintide/metreleptin combination therapy has been discontinued by the manufacturing companies because of safety concerns [249](Table **3**).

## CONCLUSION

While the development of antiobesity drugs continues to look challenging, researchers retain the hope that effective and safe medications might be on the horizon. A number of drug candidates and targets are currently being explored. Liraglutide, cetilistat, and tesofensine are already in late phases of clinical development. Thus, notwithstanding the existing skepticism about the future development of these drugs, substantial optimism persists that new antiobesity drugs will be available in the foreseeable future.

## Figures and Tables

**Table 1.** Drugs Used in the Past to Treat Obesity ^20-27,29^

Drugs Used in Past (Year of Introduction)	Year of Discontinuation	Cause of Discontinuation
Thyroid hormone	Off label use till 1980s despite toxicity	Hyperthyroidism, cardiac arrhythmias, and sudden death
Dinitrophenol (introduction in 1930)	Banned by the FDA in 1938	Dermatitis, neuropathy, agranulocytosis, visual impairment, death
Amphetamine and its derivatives (1936)	Became a schedule II drug under the Controlled Substances Act in 1971	Addiction, hypertension, myocardial toxicity
Aminorex (1965 in Europe)	1968	Chronic pulmonary hypertension resulting in high mortality
Fenfluramine + phentermine (1992)	1997	Valvular heart disease
Phenylpropanolamine(available since 1970)	2000	Hemorrhagic stroke
Rimonabant(available in UK and other European countries since 2006 for long term treatment of obesity)	EMEA withdrew marketing authorization in 2009 Failed to get US-FDA approval due to safety factors	Psychiatric disorders, depression and suicidal ideation
Sibutramine (popular antiobesity drug used since 1997)	2010	Increase in risk of major adverse cardiovascular events (a composite of non-fatal heart attack, non-fatal stroke, resuscitation after cardiac arrest and cardiovascular death)

(Abbreviation = FDA: Food and Drug Administration; EMEA= European Medicine Agency)

**Table 2.** Drugs in Early or Late Stage of Clinical Development

Current Development Status	Drug	Pharmacological Approach	Pharmaceutical Company	References
Phase 3	Cetilistat	Pancreatic lipase inhibitor	Amylin Pharmaceuticals Inc. /Takeda Pharmaceutical Co. Ltd	Kopelman *et al.* (2007) [185], Kopelman *et al.* (2010) [186], Padwal R (2008) [187]
	Tesofensine	Reuptake inhibitor of noradrenaline, dopamine and serotonin	NeuroSearch A/S	Astrup *et al.* (2008) [76], Bello *et al.* (2009) [77]
	Liraglutide	GLP1 agonist	Novo Nordisk A/S	Astrup *et al.* (2009) [127], Neary *et al.* (2009) [128]
Phase 2	Obinepitide	Analogue of PYY3-36 and PP. Agonist of neuropeptide Y2 and Y4 receptor	7TM Pharma	Neary *et al.* (2009) [128]
	Empatic	Combination of bupropion and zonisamide	Orexigen Therapeutics Inc	Valentino *et al.* (2010) [192]
Phase 1	OAP 189 (TKS-1225)	Oxyntomodulin analogue	Pfizer Inc (previously developed by Thiakis/ Wyeth)	Wyne *et al.* (2006) [135], Bloomgarden ZT (2009) [136], McGavigan *et al.* (2012) [137]

(Abbreviations : PYY3-36: peptide YY3-36; PP= pancreatic polypeptide; GLP1: glucagon- like peptide1)

**Table 3.** Drugs not Being Pursued for Future Development or for which Current Status is Not Known

Stage of Development in which the Drug Failed or was Not Pursued Further	Drug	Pharmacological Approach	Pharmaceutical Company	Comment	Reference
NDA rejected by FDA	Lorcaserin	Selective serotonin receptor agonist	Arena Pharmaceuticals Inc	High doses of the drug associated with tumor formation in rats	Smith *et al.* (2010) [83], Fidler *et al.* (2011) [84], Pollack A (2010) [85]
	Qnexa	Low doses of phentermine and topiramate	Vivus Inc	Rejected due to safety concerns including increased heart rate, teratogenic potential and psychiatric problems	Gadde *et al*. (2011) [246], Pollack A (2010) [247]
	Contrave	Fixed dose combination of bupropion and naltrexone	Orexigen Therapeutics	Deferred approval till cardiovascular safety concerns are addressed	Greenway *et al*. (2010) [240], Wadden *et al*. (2011) [242], Pollack A (2011) [243]
Phase 3	Axokine	Recombinant CNTF	Regeneron pharmaceuticals Inc	Failed due to development of antibodies against CNTF	Ettinger *et al.* (2003) [74], Pollack A (2003) [75]
Phase 2	PYY3-36 (nasal)	Neuropeptide presynaptic Y2 receptor agonist	Nastech Pharmaceutical Company	Did not meet primary efficacy endpoint	Gantz *et al*. (2007) [131]
	GI181771X	CCK1 receptor agonists	Glaxo Smithkline	Failed to demonstrate efficacy	Jordan *et al*. (2008) [116]
	AOD 9604	Growth hormone analogue	Metabolic Pharmaceuticals	Drug failed to demonstrate efficacy in phase 2b studies	Valentino *et al*. (2010) [192]
	MK0557	Neuropeptide Y 5 receptor antagonist	Merck & Co., Inc.	Failed to demonstrate efficacy	Erondu *et al.* (2006) [155]
	SCH497079	Histamine 3 receptor antagonist	Schering plough	Current status not known	ClinicalTrial.gov [92]
	MK-0493	Melanocortin 4 receptor agonist	Merck & Co., Inc.	Lack of efficacy	Krishna *et al*. (2009) [63]
	Pramlinitide and metreleptin	Combination of long-term adiposity signal eg. leptin (metreleptin, analog of human leptin) and short-term satiety signals e.g. amylin (pramlintide, analog of amylin)	Amylin Pharmaceuticals Inc, Takeda Pharmaceuticals Limited	Discountinued the project after commercial reassessment	Ravussin *et al.* (2009) [248], Tam *et al*. (2011) [249]
	*Hoodia* P57 extract	Appetite suppression	Phytopharm, Pfizer Inc,Unilever	Difficulty in synthesizing the extract in drug form, Inconclusive data on efficacy and safety	Vermaak *et al.* (2011) [94], Bray *et al*. (2007) [95]
	Famoxin	Proteolytic cleavage product of adipocyte complement-related protein (Acrp30)	Genset Corporation (acquired by Serono Co. in 2003)	Current status not known	Cheetham *et al*. (2004) [220]
	N-5984	β3 adrenoceptor agonist	Nisshin Kyorin Pharmaceuticals Co.	Current status not known	Farrigan *et al.* (2002) [202]

(Abbreviations: NDA: new drug application; FDA: Food and Drug Administration; CCK= cholecystokinin; PYY3-36: peptide YY3-36; CNTF: ciliary neurotrophic factor)
